# Supplementary material for: Maternal saliva visfatin level in term and preterm labor: A case control study
Source: PLoS One. 2023 Jul 17;18(7):e0288786. doi: 10.1371/journal.pone.0288786 (PMC10351718; doi:10.1371/journal.pone.0288786)
Supplement: Attachment — Submitted filename: Response to the reviewers.docx [file pone.0288786.s002.docx]

Dear Academic Editor,

Dear Prof. Antonio Simone Laganà,

Thank you for giving us the opportunity to submit a revised draft of our manuscript titled “**Maternal saliva visfatin level in term and preterm labor: a case control study”** to **PLOS ONE**.

We appreciate the time and effort that you and the reviewers have dedicated to providing valuable feedback on our manuscript. We are grateful to the reviewers for their insightful comments on our manuscript. Improvements throughout the manuscript were made in accordance with the reviewers’ comments. The comments were extremely valuable for improving the quality of our manuscript. We have been able to incorporate changes to reflect most of the suggestions provided by the reviewers. We have highlighted the changes within the manuscript.

Here is a point-by-point response to the reviewers’ comments.

Best regards,

Reviewers' comments:

**Comments to the Author**

1. Is the manuscript technically sound, and do the data support the conclusions?

The manuscript must describe a technically sound piece of scientific research with data that supports the conclusions. Experiments must have been conducted rigorously, with appropriate controls, replication, and sample sizes. The conclusions must be drawn appropriately based on the data presented.

Reviewer #1: Yes

Reviewer #2: Yes

Reviewer #3: Partly

Reviewer #4: Partly

**Response**: Thank you for your comment and suggestions that allowed us to greatly improve the quality of the manuscript.

2. Has the statistical analysis been performed appropriately and rigorously?

Reviewer #1: Yes

Reviewer #2: Yes

Reviewer #3: N/A

Reviewer #4: N/A

**Response**: Thank you for your comment and suggestions that allowed us to greatly improve the quality of the manuscript.

3. Have the authors made all data underlying the findings in their manuscript fully available?

The [PLOS Data policy](http://www.plosone.org/static/policies.action#sharing) requires authors to make all data underlying the findings described in their manuscript fully available without restriction, with rare exception (please refer to the Data Availability Statement in the manuscript PDF file). The data should be provided as part of the manuscript or its supporting information, or deposited to a public repository. For example, in addition to summary statistics, the data points behind means, medians and variance measures should be available. If there are restrictions on publicly sharing data—e.g. participant privacy or use of data from a third party—those must be specified.

Reviewer #1: Yes

Reviewer #2: Yes

Reviewer #3: Yes

Reviewer #4: No

**Response**: Thank you for your comment and suggestions that allowed us to greatly improve the quality of the manuscript.

4. Is the manuscript presented in an intelligible fashion and written in standard English?

PLOS ONE does not copyedit accepted manuscripts, so the language in submitted articles must be clear, correct, and unambiguous. Any typographical or grammatical errors should be corrected at revision, so please note any specific errors here.

Reviewer #1: Yes

Reviewer #2: Yes

Reviewer #3: Yes

Reviewer #4: Yes

**Response**: Thank you for your comment and suggestions that allowed us to greatly improve the quality of the manuscript.

5. Review Comments to the Author

Please use the space provided to explain your answers to the questions above. You may also include additional comments for the author, including concerns about dual publication, research ethics, or publication ethics. (Please upload your review as an attachment if it exceeds 20,000 characters)

**Reviewer #1:**

I read with great interest the Manuscript titled " Comparison of visfatin levels in saliva in women with term delivery and preterm delivery: a Case-Control Study" which falls within the aim of the Journal.

In my opinion, this topic analyzed is interesting enough to attract readers’ attention.
Methodology is accurate and conclusions are supported by the data analysis. Nevertheless, authors should clarify some points and improve the quality of the manuscript citing relevant and novel key articles about the topic:

1- I suggest a round of language revision, in order to correct few typos and improve readability.

**Response:** Thank you for your comment and suggestions that allowed us to greatly improve the quality of the manuscript. We revised the manuscript accordingly.

2- I would recommend to add further details to discuss common causes of preterm delivery, such as advanced maternal age as well as infections (authors may refer to: PMID: 25027820; PMID: 34207831).

**Response:** Thank you for your comment. We added some related explanations to the Introduction in this regard.

3- Because of these reasons, the article should be revised and completed. Tables are clear and interesting. Considering all these points, I think it could be of interest for the readers and, in my opinion, it deserves the priority to be published after minor revisions.

**Response:** Thank you for your comment and suggestions that allowed us to greatly improve the quality of the manuscript.

**Reviewer #2:**

Dear authors, congratulation

Preterm birth is a topic of great interest due to its long-term repercussions on the new born.
The potential application of the visfatin levels in saliva are of interest and deserve to be published to my opinion.

1- I would like to suggest to rephrase the title in "maternal saliva visfatin level in term and preterm labor: a case control study". I would use this rephrase for the entire paper introduction is nice methods can you explain

**Response:** Thank you for your comment and suggestions that allowed us to greatly improve the quality of the manuscript. The suggested title was replaced.

2- How did you calculate the power of the study?

**Response:** Dear reviewer, thank you for your valuable comments. At the start of study, we calculate the sample size with assuming power of 80% for the main hypothesis. At the end of study, we calculated the power with Stata for the main hypothesis and it was 90.13% as below.

3- Results well presented.

**Response:** Thank you for confirming this part of our article.

4- Conclusion correct.

**Response:** Thank you for confirming this part of our article.

**Reviewer #3**:

I read with great interest the Manuscript titled “Comparison of visfatin levels in saliva in women with term delivery and preterm delivery: a Case-Control Study”, which falls within the aim of this Journal.
In my honest opinion, the topic is interesting enough to attract the readers’ attention. Methodology is accurate and conclusions are supported by the data analysis. Nevertheless, authors should clarify some point and improve the discussion citing relevant and novel key articles about the topic.

Authors should consider the following recommendations:

1- Manuscript should be further revised by a native English speaker.

**Response:** Thank you for your comment and suggestions that allowed us to greatly improve the quality of the manuscript. We revised the manuscript accordingly.

2- In the Results section, the Authors have simply reported the p values, from which however it is not possible to deduce the real clinical relevance of the highlighted statistical significance. In order to better understand the obtained results, I suggest reporting not only the p values, but also the corresponding confidence intervals.

**Response:** Thank you for your comment. We added the 95%CI for the reported mean of visfatin by group, GDM, weight and preeclampsia in Table 3.

3- The authors have not adequately highlighted the strengths and limitations of their study. I suggest clarifying these points.

**Response:** Dear reviewer, we added some issues as limitations and strengths to the manuscript (the last paragraph of discussion).

4- Does this manuscript conform the Enhancing the QUAlity and Transparency Of health Research (EQUATOR) network guidelines? It would be mandatory to declare about this element.

**Response:** Thank you for your comment. In preparing the manuscript, we followed the STROBE Statement—Checklist of items that should be included in reports of case-control studies and this checklist was submitted to the journal.

5- I suggest to discuss, at least briefly, the risk factors associated with adverse fetal outcomes in pregnancies affected by COVID-19 (authors may refer to: PMID: 32975205; PMID: 36143264).

**Response:** Thank you for your comment. We added some related explanations to the discussion section in this regard.

**Reviewer #4**:

The story intends to clarify the mechanism inducing preterm birth from different perspectives. But there are some questions that confused me.

1- First, the authors should explain in detail the inclusion and exclusion criteria, and whether labor was initiated spontaneously. In addition, is the quantity of visfatin different in spontaneous preterm birth and iatrogenic preterm birth?

**Response:** Thank you for your comment and suggestions that allowed us to greatly improve the quality of the manuscript. We clarified the inclusion criteria and it should be highlighted that in all participants, labor had started spontaneously and we did not include iatrogenic preterm birth.

2- What’s more, the authors should point out the gestational age for sampling. Without it, the authors hardly give a conclusion that the visfatin related to the initiation of labor.

**Response:** Thank you for your valuable comment. In the comparison of visfatin mean between term and pre-term groups, the variables of GDM, preeclampsia, pre pregnancy BMI and weight gain were adjusted by binary logistic regression and gestational age has significant collinearity with groups (term/preterm) and excluded from the model. The mean of gestational age was compared between two groups and as it was reported in Table 1, its mean was 38.6±0.98 and 32.7±3.1 in control and case group.
